# Primary nutrient sensors in plants

**DOI:** 10.1016/j.isci.2022.104029

**Published:** 2022-03-04

**Authors:** Dorina Podar, Frans J.M. Maathuis

**Affiliations:** 1 Faculty of Biology and Geology and Centre for Systems Biology, Biodiversity and Bioresources (3B), Babes-Bolyai University, Cluj 400084, Romania; 2 Biology Department, University of York, YO10 5DD York, UK

**Keywords:** Biological sciences, Plant biology, Plant nutrition, Plant physiology

## Abstract

Nutrients are scarce and valuable resources, so plants developed sophisticated mechanisms to optimize nutrient use efficiency. A crucial part of this is monitoring external and internal nutrient levels to adjust processes such as uptake, redistribution, and cellular compartmentation. Measurement of nutrient levels is carried out by primary sensors that typically involve either transceptors or transcription factors. Primary sensors are only now starting to be identified in plants for some nutrients. In particular, for nitrate, there is detailed insight concerning how the external nitrate status is sensed by members of the nitrate transporter 1 (NRT1) family. Potential sensors for other macronutrients such as potassium and sodium have also been identified recently, whereas for micronutrients such as zinc and iron, transcription factor type sensors have been reported. This review provides an overview that interprets and evaluates our current understanding of how plants sense macro and micronutrients in the rhizosphere and root symplast.

## Graphical abstract

**Figure:**
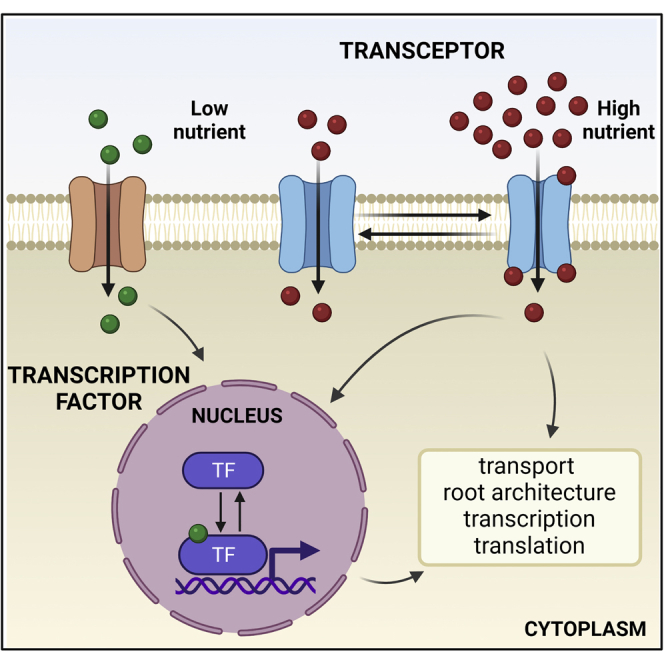

## Introduction

Plants require 14 essential nutrients (Marschner, 2012) that are taken up as minerals from the soil. Six of these are classified as macronutrients (nitrogen (N); potassium (K); calcium (Ca); magnesium (Mg); phosphorous (P); and sulfur (S)), whereas the rest is defined as micronutrient (iron (Fe), zinc (Zn), copper (Cu), boron (B), manganese (Mn), cobalt (Co), molybdenum (Mo) and nickel (Ni)). In addition to essential nutrients, soils will contain a range of further minerals, some of which can have nutritional properties but are not essential (e.g. silicon (Si) and sodium (Na)), whereas others only exert negative effects (Marschner, 2012). Examples of the latter include minerals containing (heavy) metals such as lead, cadmium, aluminium, or metalloids such as arsenic and antimony.

Uptake of these elements typically, though not exclusively, occurs at the root-soil boundary where it is mediated by specific membrane transporter proteins that often must accumulate nutrients against steep concentration gradients and from vastly variable substrates. After uptake, nutrient partitioning between intracellular compartments similarly depends on the activity of (endo)membrane transporters whereas distribution within and between various plant organs involves long-distance translocation through tissues like xylem and phloem.

**Figure 1:**
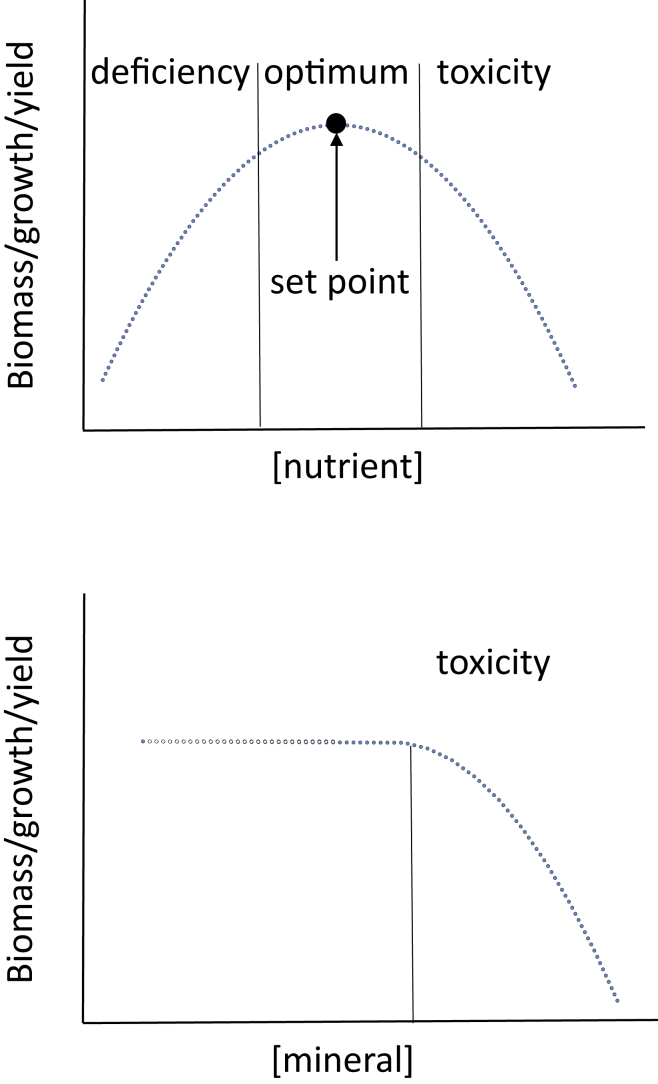
Theoretical relationship between growth parameters like biomass, growth rate and yield, and (external and/or internal) concentration of essential nutrients (top) or harmful minerals (bottom)

Adequate nutrient uptake has to be maintained in spite of large fluctuations in soil nutrient availability across space and time. Variability is owing to abiotic factors such as precipitation, temperature, wind, soil type, and soil pH. Furthermore, micro-organisms in the rhizosphere greatly impact nutrient availability. In such unstable environments, the ability to respond to changes in soil ion concentration is crucial for non-mobile plants and, consequently, plants have developed adaptive and flexible strategies to ensure sufficient nutrient acquisition. As is the case for soil nutrient levels, the optimum tissue concentrations also will critically depend on many factors such as the type of nutrient, cellular compartment, organ, etc., and is not fixed in time. The latter is exemplified by large-scale nutrient reallocation during progression through developmental stages, in particular after the switch from vegetative to regenerative growth. Whether in the context of soil solution or cellular compartments, nutrient sufficiency can be defined as an interval of concentration that is limited by deficiency on one end and toxicity on the other (Figure 1A). This range extends around some “set point” that describes the optimum concentration. Harmful elements will become noxious when a certain concentration threshold is crossed (Figure 1B). In that case, physiological and developmental responses are evoked to limit toxicity.

Whether a nutrient or toxic mineral, it is becoming increasingly clear that sophisticated signaling and transport systems are in place to ensure adequate nutrition, on the one hand, while limiting the accumulation of minerals at harmful concentrations. Thus, the spatio-temporal dynamics of mineral uptake and distribution are likely to be complex and highly controlled processes, but in all cases will critically depend on reliable information regarding mineral concentration within the plant and/or in the rhizosphere. Primary sensors that relay this information usually consist of proteins that bind specific nutrients/minerals and transduce this into a downstream signal such as a conformational change, a phosphorylation switch, or an electric signal. The ranges and set points of these sensors depend on binding affinity and may vary greatly according to locality and function. For example, sensing of external nutrients that are typically present in the rhizosphere in very dilute form may drive adaptations that optimize nutrient acquisition. In contrast, cellular nutrient homeostasis may require the measurement of nutrients in the cytoplasm and in organelles where nutrient concentrations are typically several orders of magnitude higher than those found in the rhizosphere. Differences in sensor set points are also likely to exist between organs such as shoots and roots.

Primary sensors and their downstream signaling cascades can drive rapid adaptations such as the adjustment of transport rates. This is particularly obvious for membrane located proteins that carry out both signaling *and* transport functions, the so-called transceptors. Transceptors directly modulate transport activity in response to nutrient concentration. In its most rudimentary form, this process can be modeled by a primary sensor that reports on the levels of a specific nutrient (internally, externally, or both) and couples the signaling function via a feedback loop to transport functionality. Feedback could be in the form of a conformational change within a transceptor or an intermediary compound that links to separate transporters. In either case, an almost instantaneous mechanism is presented to modulate the influx and efflux of that nutrient. Primary sensors are also indispensable for more long-term adjustment such as transcriptional and post-translational changes, modulation of metabolic pathways, or alterations in root architecture.

Where plants are concerned, primary sensors that have actually been proven to measure nutrient concentrations have only been identified in very few cases. In several more cases, we are probably “almost there” in terms of molecular identification and functional characterization. This review provides an overview that interprets and evaluates our current understanding of how plants sense macro and micronutrients in the rhizosphere and root symplast.

## Macronutrients

### Nitrogen (nitrate)

The word nitrogen derives from the Greek words “nitron” and “genesis”, which together mean “saltpetre forming”. Typically, plants contain around 1.5% dry weight nitrogen that enters the plant predominantly in the form of nitrate (${\text{NO}}_{3}^{-}$) and/or ammonium (${\text{NH}}_{4}^{+}$). The foremost function of N is to provide amino groups in amino acids, the building blocks of proteins. But N is also prolific in nucleotides as components of the ring structure of purines and pyrimidines, constituents of nucleic acids. N is also essential in the biochemistry of photosynthetic pigments and secondary metabolites (Marschner, 2012; Maathuis et al., 2014).

The multiplicity of transport systems that are involved in the uptake and distribution of N has been extensively studied, particularly in the model species *Arabidopsis thaliana* (for a review, see O'Brien et al., 2016; Rashid et al., 2018; Wang et al., 2021c). *Arabidopsis* takes up N mostly as ${\text{NO}}_{3}^{-}$ in roots that contain both low affinity transport systems (LATS, Km ∼ 1 mM) and high-affinity transport systems (HATS, Km ∼ 25 μM). LATS and HATS are encoded respectively by the nitrate transporter 1 (NRT1/NPF) and NRT2 gene families. HATS have a constitutive (HATSc) and an inducible (HATSi) component and their basic function is to provide active ${\text{NO}}_{3}^{-}$ uptake, which is energized by coupling ${\text{NO}}_{3}^{-}$ movement to the electrochemical proton (H^+^) gradient.

**Figure 2:**
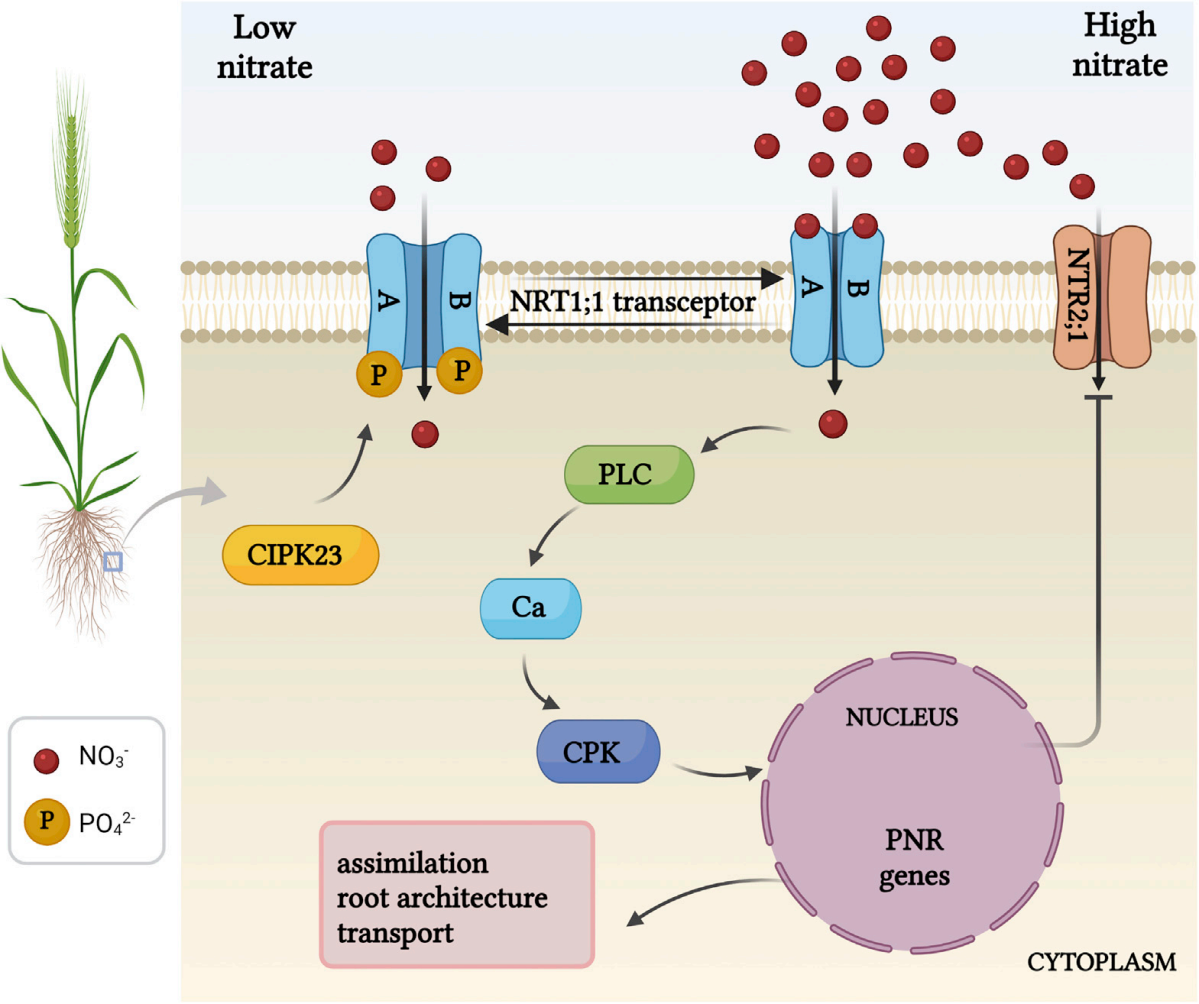
Transceptor mediated nitrate signaling In low external ${\text{NO}}_{3}^{-}$ conditions, only one of the NRT1; 1 monomers binds ${\text{NO}}_{3}^{-}$ with high affinity. This evokes conformational changes in the protein that switches NRT1; 1 to its high-affinity transport mode and provides access for the CIPK23 kinase to phosphorylate NRT1; 1. When external ${\text{NO}}_{3}^{-}$ is raised, NRT1; 1 is dephosphorylated and both NRT1; 1 monomers bind ${\text{NO}}_{3}^{-}$. The ensuing conformational change causes the monomers to interact and NRT1; 1 to switch to a low affinity/high capacity transport mode. Simultaneously, a PLC mediated Ca^2+^ signal is generated that is conveyed to the nucleus via CPKs and activates transcription of PNR genes. The PNR includes downregulation of other high-affinity ${\text{NO}}_{3}^{-}$ uptake transporters such as NRT2; 1 and physiological responses such as upregulated nitrogen assimilation and adapted root architecture. NRT, nitrate transporter; CIPK, CBL-interacting kinase; PLC, phospholipase; CPK, Ca^2+^-dependent protein kinase; PNR, primary nitrate response. Figure created with BioRender.com.

An exception to the above is NRT1;1 (also called NPF6.3 or CHL1), which can switch between HATS and LATS according to the prevalent external ${\text{NO}}_{3}^{-}$ conditions. Furthermore, NRT1;1 not only functions as transporter but also as a nitrate receptor (Figure 2); it has an important function in signaling ${\text{NO}}_{3}^{-}$ levels and is therefore called a “transceptor” (Ho et al., 2009; Table 1). Several mutational studies have shown that the transport and signaling functions of NRT1;1 are independent and can be uncoupled from each other. For example, the *chl1-9* mutant, which carries a mutation in the loop region between the 10th and 11th transmembrane domains, lacks all transport activity but is not affected in its signaling capacity (Bouguyon et al., 2015; Ho et al., 2009).

**Table 1:** Primary nutrient sensors in plants

Nutrient	Primary sensor	Type of sensor	Molecular signaling mechanism(s)	Compartment, cell type	Reference
Nitrogen (${\text{NO}}_{3}^{-}$, nitrate)	NRT1; 1 – nitrate transporter 1	Transceptor	${\text{NO}}_{3}^{-}$ binding to specific residues Phosphorylation/dephosphorylation Ca^2+^ signaling via CBL/CIPK	Root apoplast	Ho et al. (2009), Sun and Zheng (2015), Liu and von Wiren (2017), Krouk (2017), Rashid et al. (2018), Krouk (2017)
Nitrogen (${\text{NH}}_{4}^{+}$, ammonium)	AMT – ammonium transporter family isoforms	Transceptor (putative)	Phosphorylation/dephosphorylation Subunit dissociation Ca^2+^ signaling via CBL/CIPK Trafficking to and from the plasma membrane	Root apoplast	Loqué et al. (2007), Lanquar et al. (2009), Yuan et al. (2013), Wang et al. (2013), Hao et al. (2020a), (2020b)
Potassium (K^+^)	Unknown Ca^2+^ permeable channel	Transceptor	Plasma membrane hyperpolarization Ca^2+^ signaling	Cellular K^+^ in root tip division zone	Wang et al. (2021a)
	AKT1 – *Arabidopsis* K transporter 1 – inward rectifying K^+^ channel	Transceptor (putative)	Ca^2+^ signaling via CBL/CIPK Phosphorylation/dephosphorylation	Unknown	Xu et al. (2006), Wang et al. (2016)
	HAK/KUP/KT -high-affinity K transporter	Transceptor (putative)	Ca^2+^ signaling via CBL/CIPK Phosphorylation/dephosphorylation	Unknown	Feng et al. (2021), Ragel et al. (2015)
Phosphorous (${\text{PO}}_{4}^{3-}$. phosphate)	PHT1 – phosphate transporter	Transceptor (putative)	Trafficking to and from the plasma membrane	Root tip apoplast	Ham et al. (2018)
Inositol phosphate (InsP)	Transporters and regulatory proteins containing SPX domain, e.g. plasma membrane, PHO1; vacuolar, AtPHT5; 1, and OsSPX-MFS1; plasma membrane of arbuscule-containing cells MtSPX1 and MtSPX3	Transceptors and Transcription factors	Binding of InsPs such as InsP_7_ to SPX domain of transceptors (e.g. PHO1) and regulatory proteins (e.g. SPX4) that interact with transcription factors (e.g. PHRs)	Cellular InsP	Wild et al. (2016), Wege et al. (2016), Ham et al. (2018), Lv et al. (2014), Wang et al. (2021d)
Sulfur (${\text{SO}}_{4}^{2-}$, sulfate)	SULTR – sulfate transporter isoforms	Transceptor (putative)	Unknown		Wawrzyńska and Sirko (2014), Zhang et al. (2014b)
Sodium (Na^+^) and monovalent cations	GIPC (glycosyl-inositol-phosphoryl ceramide) sphingolipids	Membrane lipid	Monovalent binding lowers zeta potential Ca^2+^ signaling	Root apoplast	Jiang et al. (2019)
Zinc (Zn)	Members of F-bZIP family, e.g. AtbZIP19, AtbZIP23	Transcription factor	Zn binding to cysteine and histidine residues Transcriptional regulation	Cellular Zn in cytoplasm and nucleus	Lilay et al. (2021)
	AtMTP1 – tonoplast transporter	Transceptor (putative)	Zn binding to histidine residues between transmembrane domain 4 and 5		Tanaka et al. (2015)
	AtHMA4 – plasma membrane transporter	Transceptor (putative)	Zn binding to cysteine and histidine residues in C-terminal		Baekgaard et al. (2010)
Copper (Cu)	SBPs – Squamosa promoter binding proteins, e.g. CRR1 (lower plants) Unknown sensor (higher plants)	Transcription factor	Cu^+^ binding to highly conserved cysteine and histidine residues Binding of SBPs to copper response elements (CuREs) of target genes	Cellular Cu in cytoplasm and nucleus	Yamasaki et al. (2004), (2009), Bernal et al. (2012), Schulten et al. (2019)
Iron (Fe)	HRZ-BTS-BTSL proteins HRZ-BTS – all plants BTSL – dicot plants	Nuclear proteins	Reduced Fe^2+^ binding to the N terminal haemerythrin (Hr) domain induces protein degradation HRZ-dependent degradation of bHLH transcription factors	Cellular Fe in cytoplasm and nucleus	Kobayashi et al. (2013), Selote et al. (2015), Rodríguez-Celma et al. (2017) Hindt et al. (2017), Selote et al. (2018), Rodríguez-Celma et al. (2019)
	IDEF proteins (monocot plants)	Transcription factor	Fe binding to histidine–asparagine-proline-rich tandems IDEF binding to Fe deficiency-responsive, cis-acting element IDE1		Kobayashi et al. (2007), (2009), (2012), Ogo et al. (2008)
	IMA-FEP *proteins*	Peptides	Fe binding to a conserved asparagine-rich 17 amino acid sequence in C-terminal Association with bHLH transcription factors	Phloem; sieve cells	Grillet et al. (2018), Hirayama et al. (2018), Li et al. (2021)
Non-iron metals (Mn, Zn, Co or Cd)	IRT1 – iron-regulated transporter	Transceptor	Binding of excess non-iron metals induces phosphorylation of serine and threonine residues by CIPK and subsequent ubiquitination of IRT1 for vacuolar degradation	Cellular Fe in cytoplasm and nucleus	Barberon et al. (2011), Dubeaux et al. (2018)
	IMA-FEP *proteins*	Peptides	Binding of non-iron metals to a conserved asparagine-rich 17 amino acid sequence in C-terminal Association with bHLH transcription factors	Phloem; sieve cells	Grillet et al. (2018), Hirayama et al. (2018), Li et al. (2021)

bHLH, basic helix-loop-helix; CBL, calcineurin-B-like protein; CIPK, CBL-interacting-protein-kinase; CRR, copper response regulator; F-bZIP TFs, F-group basic leucine-zipper transcription factors; HMAs, heavy metal ATPases; HRZ-BTS-BTSL, haemerythrin motif-containing Really Interesting New Gene (RING)- and Zinc-finger proteins/BRUTUS/BRUTUS(-like) [BTS(L)]; IDE1, Fe deficiency-responsive, cis-acting element; IDEFs, Iron deficiency-responsive element-binding factors; IMA-FEPs, Iron man-Fe-uptake inducing peptides; MTPs, metal tolerance proteins; PHR, phosphate starvation response; SPLs, Squamosa promoter binding protein like; SPX, (SYG1/PHO81/XPR1) protein.

NRT1;1 signaling function critically depends on phosphorylation/dephosphorylation of a specific residue, Thr101 (Bouguyon et al., 2015; Ho et al., 2009; Wang et al., 2021c). The phosphorylation state of this residue impacts NRT1; 1 signaling events that vary in scale and time (Figure 2). Firstly, NRT1; 1 transport is directly affected; when the ambient ${\text{NO}}_{3}^{-}$ concentration is low, Thr101 phosphorylation disrupts dimerization of NRT1; 1 (Sun and Zheng, 2015) and this establishes a high-affinity mode of transport. Vice versa, when external nitrate is abundant, dephosphorylation of NRT1;1 leads to the formation of a dimer that functions as a low-affinity nitrate transporter. However, it gets better still: ${\text{NO}}_{3}^{-}$ dependent signaling not only modulates ${\text{NO}}_{3}^{-}$ uptake via NRT1; 1 affinity, it also impacts on a raft of more long term and downstream responses such as the “primary nitrate response” (PNR) (Bouguyon et al., 2015; Liu and Tsay, 2003; Muños et al., 2004; Wang et al., 2021d). The PNR includes transcriptional regulation of other ${\text{NO}}_{3}^{-}$ transporters and N-assimilatory genes. PNR activation depends on Ca^2+^ signaling signaling (Liu and von Wirén, 2017; Rashid et al., 2020) and requires stabilization of the dimeric (low affinity) configuration of NRT1; 1. Recent work suggests that the necessary Ca^2+^ signals occur via a molecular switch that is made up of NRT1; 1 and the Ca-permeable cyclic nucleotide-gated channel 15 (CNGC15) (Wang et al., 2021c). Using split ubiquitin and BiFC assays, it was shown that CNGC15 can associate with NRT1; 1 in the membrane. Electrophysiology showed that formation of the CNGC15:NRT1; 1 complex blocks the channel function in an ${\text{NO}}_{3}^{-}$ dependent manner: no Ca^2+^ current appeared in the absence of nitrate, whereas perfusion with a 1 mM ${\text{NO}}_{3}^{-}$ solution produced a small Ca^2+^ current. The current amplitude was further increased when the external ${\text{NO}}_{3}^{-}$ concentration was raised to 10 mM. In all, this scheme ensures that in conditions of plentiful ${\text{NO}}_{3}^{-}$ the CNGC15:NRT1; 1 complex dissociates, which restores CNGC15 function and hence generates a Ca^2+^ signal that initiates downstream processes such as the PNR. A prime example of the PNR in action is the repression of the high-affinity transporter NRT2; 1 under low-affinity ${\text{NO}}_{3}^{-}$ conditions (Figure 2), which is instigated by NRT1.1 dependent signaling, involves Ca^2+^-dependent kinases (CPKs) and the transcription factor NLP7 (NIN-like protein 7) (Wang et al., 2021c).

The third way in which phosphorylation of Thr101 affects N nutrition is by altering the root architecture. This is based in the capability of NRT1; 1 to not only transport ${\text{NO}}_{3}^{-}$ but also the growth hormone auxin that is responsible for lateral root development (Asim et al., 2020). Under low ${\text{NO}}_{3}^{-}$ conditions, NRT1; 1 represses lateral root development by promoting basipetal auxin transport. This lowers auxin levels in the root apical meristem and lateral root primordium thus reducing lateral root growth (Asim et al., 2020; Bouguyon et al., 2015; Krouk et al., 2010).

One intricate piece of the puzzle concerns the mechanistic link between ${\text{NO}}_{3}^{-}$ sensing and signaling by NRT1; 1. Sensing occurs via binding of the substrate at the external face, whereas signaling critically depends on phosphorylation of Thr101, which is located on the opposite side of the protein, facing the cytoplasm. Detailed structure-function studies show that the separate protomers (A and B) that make up the NRT1;1 dimer are not the same (Rashid et al., 2018); for instance ${\text{NO}}_{3}^{-}$ binding in protomer A involves Thr360 and His356, but in protomer B, it is Arg45 and His356. Furthermore, the ${\text{NO}}_{3}^{-}$ binding site of protomer A has a higher affinity than that of protomer B and thus is the only monomer that binds NO_3_^-^ in high-affinity conditions, whereas in low-affinity conditions, both protomers will bind ${\text{NO}}_{3}^{-}$. Based on the NRT1; 1 structure (Sun and Zheng, 2015), computational molecular modelling was carried out (Rashid et al., 2018), showing that in protomer A, one of the alpha helices shows considerable flexibility and connects the ${\text{NO}}_{3}^{-}$ binding site with the phosphorylation site, enabling allosteric communication between the two. In contrast, a more rigid architecture in protomer B prevents such interaction. Consequently, high-affinity binding of ${\text{NO}}_{3}^{-}$ to protomer A causes asymmetric conformational changes that force the dimer to uncouple and toggles the transporter from its low to its high-affinity mode. Furthermore, they affect the transmembrane pathway for ${\text{NO}}_{3}^{-}$ transport and make Thr101 accessible for phosphorylation (Rashid et al., 2018). The latter allows the CBL-interacting-protein-kinase 23 (CIPK23) to phosphorylate Thr101 but only at the high-affinity A monomer. Stabilization of the phosphorylated state requires activation and binding of CIPK23 by calcineurin-B-like 9 (CBL9) that itself is activated by Ca^2+^ (Krouk, 2017). In contrast, high external ${\text{NO}}_{3}^{-}$ concentrations ensure that the Thr101 residue remains deeply buried in a hydrophobic pocket surrounded by residues from the dimer interface and therefore is not available for CIPK23 in either monomer (Rashid et al., 2018). Whereas flipping between transport modes initially may be entirely owing to conformational changes that promote Thr101 phosphorylation, stabilization of the phosphorylated protein may require Ca^2+^ signaling to activate the CBL9-CIPK23 cascade (Krouk, 2017).

### Nitrogen (ammonium)

The second main form in which plants take up nitrogen is as ammonium (${\text{NH}}_{4}^{+}$). ${\text{NH}}_{4}^{+}$ is the dominant from of nitrogen in non-aerobic (reductive) environments such as submerged fields and when the supply of ${\text{NO}}_{3}^{-}$ is low. The uptake of ${\text{NH}}_{4}^{+}$ is particularly preferred when it is present at micromolar concentrations because at higher levels ${\text{NH}}_{4}^{+}$ rapidly becomes toxic (Liu and von Wirén, 2017).

As was the case for nitrate, ammonium triggers a diverse range of physiological and morphological responses that include transcriptional regulation, changes in redox status, altered metabolism, and morphological adaptation, particularly of roots. In many of these responses, it is likely that ${\text{NH}}_{4}^{+}$ acts as a signaling agent.

The uptake and distribution of ${\text{NH}}_{4}^{+}$ is predominantly mediated by members of the ammonium transporter (AMT) family (Hao et al., 2020a; Liu and von Wirén ,2017). Although conclusive evidence has yet to appear, it is likely that several AMT isoforms function as transceptor, analogous to the NRT1; 1 nitrate transporter described above (Table 1). As is the case for NRT1; 1, phosphorylation plays a critical role in the allosteric regulation of AMT transporters (Loqué et al., 2007). AMT isoforms such as AMT1; 1 function as trimers with each monomer containing a threonine phosphorylation site (Thr460) in its C-terminal (Yuan et al., 2013). Under low ${\text{NH}}_{4}^{+}$ conditions, the C termini are connected to the pore of neighboring monomers and ${\text{NH}}_{4}^{+}$ transport is allowed (; Hao et al., 2020a; Liu and von Wirén, 2017). However, increased ambient [${\text{NH}}_{4}^{+}$] causes phosphorylation of Thr460 and other conserved residues (Lanquar et al., 2009; Loqué et al., 2007), which breaks the monomer association and hence inhibits ${\text{NH}}_{4}^{+}$ uptake. Mutations at similar (Thr460) positions in other AMT isoforms led to comparable effects, pointing to a universal mode of AMT regulation (Hao et al., 2020b; Wu et al., 2019; Yuan et al., 2013). Furthermore, when various ${\text{NH}}_{4}^{+}$ assimilation products were tested, none of them impacted on Thr460 phosphorylation, which suggests that this pathway responds specifically to external [${\text{NH}}_{4}^{+}$] (Hao et al., 2020b).

An alternative signaling mechanism may pertain to the putative transceptor AMT1; 3 in order to prevent toxic levels of ${\text{NH}}_{4}^{+}$ uptake (Wang et al., 2013). At low ${\text{NH}}_{4}^{+}$ supply, AMT1; 3 resides in the plasma membrane where it participates in ${\text{NH}}_{4}^{+}$ uptake. But after the addition of ${\text{NH}}_{4}^{+}$ AMT1; 3 protein immediately disappears from the plasma membrane and is internalized via endocytosis (Hao et al., 2020a; Wang et al., 2013). Thus, in both the cases of AMT1; 1 and AMT1; 3, there is tantalizing evidence of transceptor functionality but definitive confirmation of this idea is needed.

### Potassium

Potassium (K) is the most abundant cation in most plants and an essential cofactor for many enzymes. Furthermore, cytoplasmic K^+^ helps balance negative charges on proteins, whereas vacuolar K^+^ plays an important role in turgor provision and water homeostasis (Maathuis, 2009). Owing to its immobility, bioavailable K^+^ is often low in soils and typically in the micromolar range but can also reach millimolar levels. There are two main transport systems that deal with variable external K^+^ supply; first, inward rectifying K^+^ channels such as the *Arabidopsis* K transporter 1 (AKT1) with a millimolar Km that mediate passive K^+^ uptake and primarily function in low affinity conditions (Nieves-Cordones et al., 2014). Secondly, carriers from the high-affinity K transporter (HAK/KUP/KT) family are proton coupled and hence provide active K^+^ uptake with a Km in the micromolar range. HAKs are active in high-affinity conditions and are the sole K^+^ uptake mechanism when external K^+^ concentrations drop below ∼10 μM (Nieves-Cordones et al., 2014).

As was seen for nitrogen, it is very likely that K^+^ itself is an important signal in maintaining nutritional homeostasis. Changes in environmental K^+^ levels cause short- and long-term adaptation and regulation; raised K^+^ levels do not lead to rapid root proliferation as in the case of ${\text{NO}}_{3}^{-}$ and ${\text{NH}}_{4}^{+}$, but low [K^+^] conditions do inhibit the growth of the primary root and first-order lateral roots while promoting the development of higher order branching (Sustr et al., 2019). Short-term transcriptional and post-transcriptional regulation of transport has also been documented: low [K^+^] rapidly (within 1 h) induces transcription of high-affinity transport systems such as HAK5 in *A. thaliana* (Feng et al., 2021) to maintain uptake from micromolar concentration. The above is paralleled by a molecular switch analogous to that seen for NRT1; 1-mediated ${\text{NO}}_{3}^{-}$ uptake; cooperation of several CBL isoforms activates CIPK23, which phosphorylates HAK5 and the ensuing conformational changes not only increase HAK5 affinity for K^+^ but also enlarges HAK5 Vmax (Ragel et al., 2015). In contrast to *HAK5, AKT1* transcript level is largely impervious to ambient K^+^ conditions but activity of this channel is post-translationally upregulated in response to low [K^+^]. In *Arabidopsis*, this requires interaction of CBL1 and CBL9 with CIPK23. CIPK23-mediated phosphorylation of AKT1 increases K^+^ uptake via this channel (Wang et al., 2016). However, in these low [K^+^] conditions, AKT1 can function as a K^+^ leak (Geiger et al., 2009), so a second mechanism therefore downregulates AKT1 activity and antagonizes its CIPK23-based upregulation. This happens via K channel 1 (KC1) that is a “silent” channel subunit trafficked from the endoplasmic reticulum to the plasma membrane during low [K^+^] conditions, and leads to the formation of non-functional channels via association with AKT1 subunits (Geiger et al., 2009). This prevents cellular K^+^ loss during conditions where a steep outward K^+^ gradient prevails (Geiger et al., 2009; Wang et al., 2016).

The above strongly indicates a regulatory and signaling function of K^+^ but the initial upstream events remained elusive, particularly regarding the activation of the CBL proteins. Elegant work from Yi Wang and colleagues (Wang et al., 2021a) using a FRET-based K^+^ reporter (GEPII) has helped to make progress in this regard; work showed that lowering external [K^+^] from 5 to 0.1 mM causes a rapid (within minutes) reduction of cellular [K^+^] but only in the cell division zone of the root tip, whereas more mature parts of the root maintained their cytoplasmic [K^+^]. This suggests a localized, cell-specific K^+^ signal that reports on the internal K^+^ status. Interestingly, the dynamics of the [K^+^] changes largely overlapped with those of a low-[K^+^] induced Ca^2+^ signal (Wang et al., 2021a) that is evoked on a time scale of seconds after external [K^+^] is reduced from millimolar to micromolar levels. The use of the Ca^2+^ channel blocker lanthanum provides evidence that the Ca^2+^ signal is upstream of, and essential for, the generation of the K^+^ signal.

These findings provide a promising scenario of how plants sense low K^+^ conditions; an initial Ca^2+^ signal leads to activation of K^+^ channels and a subsequent K^+^- efflux from specific root tip cells (Table 1). The ensuing drop in internal [K^+^] would be the driver of subsequent signaling episodes. The Ca^2+^ signal, via various CBL isoforms, activates CIPK23, which, in turn, adjusts HAK (Ragel et al., 2015) and AKT (Xu et al., 2006) activity to low K^+^ conditions. In terms of the role of calcium and CIPK phosphorylation, there are clear parallels with the processes discussed for nitrogen but fundamental differences as well; in the case of ${\text{NO}}_{3}^{-}$, it has been firmly established that the transceptor reports the *external* nutrient concentration. In contrast, although a reduction in ambient [K^+^] must form part of an initial signal, work on K^+^ suggests that *internal* sensing is crucial in subsequent signaling events. Reporting on the intracellular status of nutrients makes physiological sense and may serve a different purpose to that seen for nitrate signaling; in the case of nitrate, optimizing *nutrient acquisition* is the main objective whereas the aim of internal sensing, such as described for K^+^, pertains to optimizing *nutrient homeostasis*. The latter includes important functions such as maintaining cytoplasmic [K^+^] or partitioning of K^+^ into vacuoles (e.g. Ahmad et al., 2016), and hence affects the nutrient use efficiency. Nevertheless, additional, as yet unknown, mechanisms for sensing external [K^+^] are likely to exist, which could include using membrane lipids as primary sensors such as described below for Na^+^.

Further open questions concern the signaling events that lie upstream of the Ca^2+^ signal; it has been suggested that a low [K^+^]-induced hyperpolarization could lead to Ca^2+^ influx (Wang et al., 2021a), but this seems unlikely since it lacks all specificity (many processes can cause a hyperpolarization). Other low K^+^ conditions, such as the onset of salinity, actually lead to a depolarization (e.g Maathuis et al., 2014). It is more likely that proteins with specific K^+^ binding properties trigger a Ca^2+^ signal. Precedents for these are found in the form of K^+^-dependent enzymes like pyruvate kinase or the K^+^ binding proteins (Kbps) found in the bacterium *Escherichia coli* (Ashraf et al., 2016). K^+^ binding in these proteins is highly specific and ensured via coordination with four or five negative oxygens from amino-acid side groups or water. Alternatively, AKT and/or HAK transporters may have transceptor functionality that relies on K^+^ binding in the cytoplasmic rather than apoplastic compartment.

### Phosphorous

The physiological functions of phosphorous (P) are widespread by being a component of phospholipids, P is responsible for the maintenance of cell membrane integrity. As part of the backbone, P is crucial for the biochemistry and functioning of nucleic acids, whereas inorganic P (Pi) is an essential component of bioenergetics. In the form of Pi, P provides a main mechanism for the regulation of proteins via phosphorylation/dephosphorylation (Maathuis, 2009).

Plants take up P primarily in the form of inorganic Pi (PO_4_^3-^). Pi deficiency is common in many habitats and frequently limits the plant growth and crop yield. Optimizing P use efficiency is of utmost importance, moreover since P fertilizer is a limited resource. In response to low Pi, plants adjust their root system architecture to maximize Pi acquisition from layers containing higher levels of Pi (Ham et al., 2018; Liao et al., 2001), which typically involves inhibition of primary root elongation in favor of lateral root formation. Other common responses are increased formation and elongation of root hairs and mycorrhizal symbioses. Some of these adaptations appear to depend on changes in external Pi levels (Williamson et al., 2001), suggesting a need for primary Pi sensors that face the rhizosphere, whereas others are more systemic and rely on cellular P status.

Work on *A. thaliana* roots has shown that local sensing of external [Pi] occurs particularly in the root tips, though the mechanistic details are not understood (Ham et al., 2018; Zhang et al., 2014b). Several early components have been identified that are involved in Pi sensing such as LPR (Low Phosphate Root) and PDR (Phosphate Deficiency Response) genes (Svistoonoff et al., 2007). LPRs bind to PDRs in response to low Pi, an interaction that is under control of transcription factor STOP1 (Sensitive to Proton Rhizotoxicity 1; Balzergue et al., 2017), and induces malate efflux via ALMT1 (Aluminium Activated Malate Transporter 1). The ferroxidase activity of the cell wall located LPRs, in combination with malate, leads to the generation of reactive oxygen species (ROS) and subsequent callose formation (Müller et al., 2015). The callose interrupts plasmodesmal trafficking of transcription factors that normally maintain stem cell activity and hence inhibit primary root growth (Balzergue et al., 2017; Ham et al., 2018).

Although the above signaling mechanism has been studied in great detail, it remains unanswered how external [Pi] is initially sensed and how that information would couple rhizosphere Pi levels to STOP1 activation. Overall P homeostasis involves the high-affinity Pi uptake carrier PHT1 whose activity depends on the net effect of trafficking between endomembranes and plasma membrane, and vacuolar degradation (Ham et al., 2018). PHT1 is a good candidate to function as a transceptor-type sensor and is a close homologue of the yeast PHO84 transceptor (Popova et al., 2010; Schothorst et al., 2013). Unfortunately, separating a putative PHT1 signaling function *per se* from its transport function and many pleiotropic effects on P homeostasis has as yet been unsuccessful (Ham et al., 2018).

In contrast, clearer insights have been obtained about potential primary sensors that monitor *internal* P status (Wild et al., 2016). These do not rely on internal [Pi] but instead report on inositol polyphosphates (InsPs) such as InsP_7_. Multiple studies provide evidence that so-called SPX (SYG1/PHO81/XPR1) proteins can bind InsPs and function as primary sensors in a range of organisms (Wild et al., 2016). SPX proteins are involved in both P transport and its regulation. An example is the binding of InsP_7_ to the SPX protein PHO1 that catalyzes the cellular release of Pi into the pericycle during P replete conditions. Subsequent loading of P into the xylem ensures adequate translocation of P to the shoot (Ham et al., 2018). In parallel, binding of InsP_7_ to other SPX proteins such as SPX4 in rice, causes an interaction with phosphate starvation response (PHR) transcription factors, preventing migration of PHRs to the nucleus. Thus, the SPX-PHR protein–protein interaction prevents the expression of P-starvation response genes (Lv et al., 2014).

Transporters at the vacuolar membrane that mediate vacuolar Pi accumulation may undergo similar regulation; in yeast, the vacuolar transport chaperone (VTC) complex mediates vacuolar P uptake after association of InsP_7_ with its SPX domain (Yang et al., 2017). Plant vacuole P transporters like AtPHT5; 1 also contain SPX domains, suggesting that direct sensing of cytoplasmic InsP is crucial for their activity (Wang et al., 2021d; Yang et al., 2017). However, the exact nature of the potential InsP-AtPHT5 interaction remains to be investigated.

Regulation of vacuolar P may also be controlled by more upstream SPX proteins; In rice, *OsSPX-MFS1* (an ortholog of *AtPHT5;1*) transcript level is a target of the microRNA miR827 (Lin et al., 2010; Lv et al., 2014; Yang et al., 2017), which itself is upregulated by the PHR2 transcription factor. Initial sensing function in this pathway could stem from PHR2 interaction with a cytoplasmic SPX protein (Lv et al., 2014; Wang et al., 2021d).

More recent literature furthermore suggests that SPX proteins may also control Pi influx through mycorrhizal fungi; expression of SPX1 and SPX3 in *Medicago trunculata* is restricted to arbuscule-containing cells of the root after the establishment of the mycorrhizal symbiosis. In these specific cells, biosynthesis of the hormone strigolactone is upregulated, which promotes fungal branching and increased root colonization (Wang et al., 2021b).

### Magnesium

Magnesium (Mg) is essential to plants primarily because of its important role in chlorophyll. In addition, Mg activates many enzymes and balances negative charges, particularly those of nucleotides. High levels of Mg in the soil can be toxic, and the work by Tang et al. (2015) suggest an important role of vacuolar Mg deposition to avert toxicity, possibly mediated by a CBL-CIPK network that is, in turn, activated by an Mg-induced Ca signal. However, no primary Mg sensor are known.

### Calcium

Calcium (Ca) is very abundant in the lithosphere but deficiency can occur after severe weathering and leaching in combination with low soil pH (Maathuis, 2009; Thor, 2019). Calcium fulfills important structural and signaling functions. It is found at high levels in the plasma membrane and cell wall. Ubiquitous in its use as the secondary messenger, it is no surprise that hundreds of proteins can reversibly bind Ca. Calcium binding is typically via so-called EF hands, which have a helix-loop-helix topology to specifically coordinate Ca^2+^. Binding-debinding of Ca^2+^ at EF hands in turn causes conformational changes that translate into altered enzyme activity (Dodd et al., 2010). Thus, there is a clear and well-proven mechanism for Ca as a secondary signaling moiety, with numerous proteins sensing cytoplasmic Ca^2+^ concentration. Nevertheless, sensing and regulatory mechanisms related to Ca as a nutrient are as yet to be discovered and, ironically, research efforts in this area may be severely hampered by the overwhelming number of Ca sensors that is typically present in a cell but are not related to sensing of Ca as a nutrient.

### Sulfur

In plants, sulfur (S) acquisition is in the form of sulfate (${\text{SO}}_{4}^{2-}$) and mediated by SULTR-type transporters. Within cells, S is primarily found in reduced form as part of the amino acids cysteine and methionine. In cysteine, sulfhydryl (–SH or thiol) groups partake in the formation of covalent sulfur bridges and hence the regulation of protein activity (Maathuis, 2009). Sulfur is also a mobile redox carrier in compounds like glutathione and, furthermore, it appears in membranes in the form of sulfolipids, particularly in thylakoid membranes.

In plants, S deficiency does involve the transcription factor, Sulfur LIMitation1 (SLIM1), which promotes up-regulation of transporter, assimilation, and other S-related genes under sulfate starvation (Wawrzyńska and Sirko, 2014). However, SLIM1 does not bind S, and upstream components such as the actual S sensor of this pathway are unknown. It has been suggested that some members (e.g. SULTR1; 2) of the SULTR family could function as the transceptor (e.g. Zhang et al., 2014a) but as yet there is no hard evidence to support this notion. In contrast, several sulfate transporters in *Saccharomyces cerevisiae* yeast (Sul1 and Sul2) do function as S-starvation responsive transceptors (Kankipati et al., 2015).

### Sodium

Sodium (Na) is not essential for most plants apart from a subgroup of C4 plants that uses Na^+^ to drive the uptake of pyruvate into chloroplasts by a Na^+^-pyruvate cotransporter (Furumoto et al., 2011). However, Na has been shown to be beneficial and “nutritious” in many plants, in particular when K is in short supply (Hartley et al., 2020; Maathuis et al., 2014; Subbarao et al., 2003). At higher external levels, Na causes salinity stress that limits agricultural production in many areas of the world (Flowers and Colmer, 2008). The presence of Na lowers the soil water potential, and hence creates osmotic stress, whereas symplastic accumulation causes ionic toxicity. These two processes are interlinked since avoidance of osmotic stress is often achieved via uptake of “cheap” osmotica in the form of Na^+^ and Cl^−^ ions, a process that requires careful control to prevent ion toxicity.

Sensing of [Na^+^] is relatively well understood in animals and typically related to Na^+^ channel activity. For instance, serum Na^+^ levels are kept in a narrow range (∼135–145 mM) by using primary sensors in the glial cells of the brain. The sensors themselves consist of Na^+^ channels whose gating (activity) is controlled via the binding of Na^+^ on the luminal side of the channel (Rose and Verkhratsky, 2016; Watanabe et al., 2006). These channels activate when [Na^+^] in the blood exceeds around 140 mM, driving a large flux of Na^+^ from serum into glial cells. In nematodes, a similar mechanism reports on excessive ambient Na^+^ levels via “transmembrane channel like” (TMC) proteins located in the cilia. Like their glial cell counterparts, these channels activate when external [Na^+^] becomes higher than around 150 mM and their activation catalyzes an avoidance reaction of the nematode (Chatzigeorgiou et al., 2013). A third example of putative sodium transceptors are the epithelial Na^+^ channels (Enacs) in taste buds that generate a Na^+^-dependent depolarization, and commensurate neurotransmitter release, which is proportionate to the amount of Na^+^ that is present in your mouth (Bigiani, 2020).

**Figure 3:**
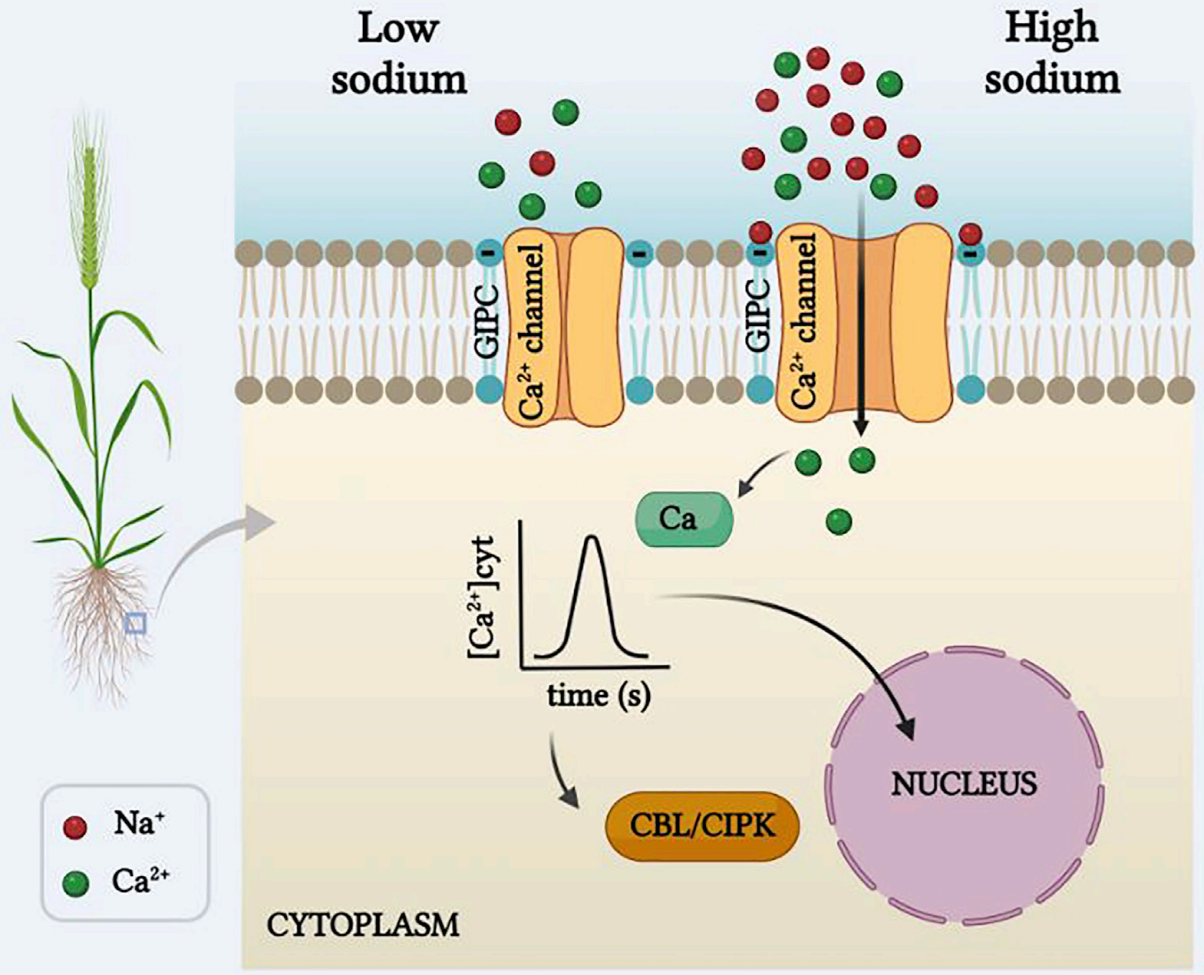
Monovalent cation signaling When the concentration of external Na^+^ (or other monovalent cations) is low, no or little binding of Na^+^ to the negative charge of the primary sensor GIPC sphingolipid occurs. High levels of Na^+^ cause electrostatic interaction between GIPC and the cations, which affects the membrane surface potential. The change in membrane voltage could promote opening of voltage dependent Ca^2+^-permeable channels and evoke a subsequent Ca^2+^ signal. The latter is translated into transcriptional regulation and well-characterized Ca^2+^-based signaling cascades such as the SOS Na^+^ extrusion pathway, activated by the CBL-CIPK interaction (Zhu, 2016). GIPC, glycosyl inositol phosphoryl ceramide; CBL, calcineurin binding protein like; CIPK, CBL-interacting kinase. Figure created with BioRender.com.

No Na^+^-selective ion channels have been identified in plants, making it very unlikely that plant Na^+^ sensing works in a similar fashion to that in animals. In fact, the Na^+^ sensing process that has been described for plants relies on cation binding to membrane lipids rather than proteins (Figure 3). The evidence for this is based on a mutant screen that exploited the long-known observation that a raise in external NaCl generates a rapid (within seconds) Ca^2+^ signal. Extensive experimentation by Jiang et al. (2019) led to the identification of a T-DNA *Arabidopsis* mutant (called “monocation-induced-[Ca^2+^]i-increases-1” or ‘MOCA1’) that was defective in salt-induced Ca^2+^ signals. Surprisingly, MOCA1 turns out to be an enzyme that transfers a glucuronosyl sugar to glycosyl-inositol-phosphorylceramide (GIPC) sphingolipids in the plasma membrane. The glycosylation of sphingolipids by MOCA1 creates negative sites at which monovalent cations can bind. Electrostatic binding of cations to the negative sites will reduce the surface (or “zeta”) potential and thus alters the local transmembrane potential, a parameter that affects the activity of voltage-dependent ion channels. The model suggests that neutralizing the negative GIPC groups by positive Na^+^ ions in the vicinity of voltage-dependent Ca^2+^ channels can promote channel opening. Thus, this scenario couples binding of (monovalent) cations on the outer leaflet of the membrane to the gating of (voltage-dependent) Ca^2+^ permeable channels and in that manner evokes a Ca^2+^ signal and further downstream signaling (Figure 3).

If this narrative is correct, it would be unique in several ways; firstly, previous electrophysiological studies have shown that although surface potentials affect ion channel properties, the effects are usually marginal and concern channel conductance rather than channel gating (e.g. Green and Andersen, 1991). In cases where surface and zeta potentials affect gating this is typically via binding and de-binding of a charged lipid (such as the binding of phosphatydyl inositol bisphosphate to Kv7.1 channels; Zaydman et al., 2013), rather than binding of (monovalent) charges to lipids. In these cases, lipid binding couples the voltage sensing function to opening of the gate in the channel pore domain via conformational changes (e.g. Poveda et al., 2014) and there is very little evidence of altered zeta potentials actually gating an ion channel. Another issue is the lack of specificity; since this signaling mechanism is initiated by electrostatic binding of any monovalent cation, it is unlikely to show any sodium/salt selectivity. However, this is not necessarily a problem because in natural environments monovalents other than Na^+^, such as K^+^ or Li^+^, are unlikely to reach “salinity” concentrations; the Li^+^ level in all natural soils is very low (Aral and Vecchio-Sadus, 2008), whereas that of K^+^ is unlikely to exceed a few millimolar (Maathuis, 2009).

A further intriguing finding is the calorimetry assays to determine dissociation constants for monovalent cation binding to GIPCs. A Kd value of only ∼0.3 mM was found for Na^+^ and similar values for K^+^ and Li^+^. Saline conditions typically imply NaCl levels that are a hundred or even a thousand-fold higher than 0.3 mM, which suggests that (a) the dynamic range for salinity sensing by GIPCs is completely out of kilter and (b) that relatively modest variation in external K^+^ (say a raise from 0.1 to 1 mM) would evoke a salinity stress response. Molecular identification of the relevant Ca^2+^-permeable channel could help to further unravel this enigmatic signaling process.

In addition to GIPCs, plants may contain proteins that have Na^+^ binding sites and are involved in internal sensing and/or signaling. Cytoplasmic Na^+^ may need monitoring to limit its build-up and to ensure adequate deposition in the vacuole. Alternatively, excess Na^+^ may need delivery to the xylem for long-distance transport to the shoot (Maathuis et al., 2014). In theory, these functions could be mediated by any protein whose activity depends on binding of intracellular Na^+^. Many examples have been found in mammals: For instance, the activity of the mammalian protease thrombin is modulated allosterically by Na^+^ with a Kd of around 20 mM (Huntington, 2008), whereas G-protein-coupled adenosine and dopamine receptors have conserved Na^+^ binding sites that modulate their affinity to agonists (Selvam et al., 2018). In these proteins, Na^+^ is typically coordinated by carbonyl oxygens from a range of residues such as the consensus motif DxR/KxxH of Na^+^-activated K^+^ channels (Zhang et al., 2010). Unfortunately, the large degree of motif redundancy makes homology searches problematic and non-specific; for example, a search in the *Arabidopsis* proteome using the DxR/KxxH sequence yields more than 1,200 candidate proteins that include transporters, transcription factors, and kinases (Maathuis et al., 2014).

## Micronutrients

### Zinc

Zinc (Zn), unlike iron and copper, is redox neutral and is reactive as a Lewis acid in biological reactions (Baś et al., 2017). Because of these features, zinc plays key roles as a structural, catalytic, and regulatory component of many enzymes (Andreini et al., 2006; Broadley et al., 2007; Capdevila et al., 2016; Kambe et al., 2015; Sinclair and Krämer, 2012; Stefanidou et al., 2006). As a structural and regulatory component, Zn is a constituent of many transcription factors (TFs) that contain “zinc finger” structural motifs that promote TF-DNA binding (Eide, 2020). Zinc fingers are also instrumental in many protein–protein interactions (Brown, 2005; Cassandri et al., 2017; Han et al., 2020; Kluska et al., 2018). Zn is the only metal represented in all six enzyme classes: oxidoreductases, transferases, hydrolases, lyases, isomerases, ligases (Broadley et al., 2007).

Zn uptake, efflux, and compartmentation has to be strictly controlled. However, not the total Zn in the cell, which in plants ranges from 0.3 to 3 mM, but the free cytosolic [Zn^2+^] together with the Zn labile pool (Zn bound to nicotianamine (NA), histidine, glutathione, phytochelatins, or phosphate) are the key factors involved in Zn homeostasis. In most cases, regulation (for example of transport) to maintain homeostasis is typically initiated by cytoplasmic Zn sensors that consist of TFs with Zn-specific binding sites. Prokaryotic examples include TFs that report on excess Zn such as ZntR in *E. coli*, or those that report on Zn deficiency like Zur in *Bacillus subtillis* (Gaballa et al., 2002; Mikhaylina et al., 2018). Orthologous systems are found in yeast (Zap1, Loz1) and mammals (MTF1) (Eide, 2009; Günther et al., 2012).

**Figure 4:**
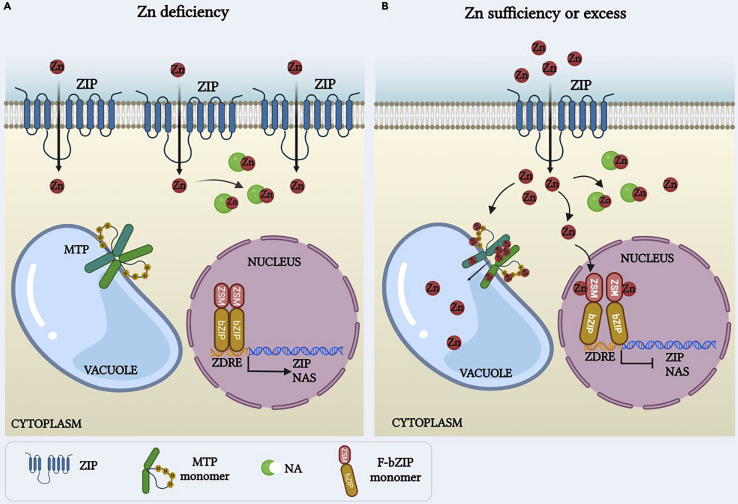
Zinc sensors in plant cells (A) When the concentration of external Zn^2+^ is low, the Cys/His-rich zinc sensor motif (ZSM) of bZIP transcription factors is free of ions. Thus, the bZIP TFs adopt a conformation that favors their binding to ZDRE promoter elements of genes coding for uptake and mobilization of Zn^2+^. (B) When the concentration of Zn^2+^ is sufficient, the metal ions within the nucleus bind to the ZSM of bZIP TFs, causing a conformational change that prevents their binding to DNA. Expression of genes involved in Zn uptake and mobilization is therefore drastically reduced. Excess Zn^2+^ in the cytoplasm is sensed by the tonoplast localized metal tolerance proteins (MTPs) through their His-rich loop. Zn^2+^ binding initiates MTP activity and promotes vacuolar sequestration of excess Zn. Figure created with BioRender.com.

Cellular Zn sensing in plants was only recently characterized and is similar to the above-mentioned systems; the local Zn status is monitored by F-group basic leucine-zipper (F-bZIP) TFs (Assunção et al., 2010). Activated TF dimers can bind to promoter regions of relevant genes. For instance, Zn deficiency causes bZIPs to bind to Zinc Deficiency Response Elements (ZDREs) of target genes that code for proteins involved in uptake and distribution of Zn. The latter includes uptake via Zinc-regulated/Fe-regulated-like proteins (ZIP/IRT-like) such as ZIP4 (Assunção et al., 2010) and ZIP9 (Inaba et al., 2015) or the nicotianamine synthases (NAS2, NAS4) (van de Mortel et al., 2006; Weber et al., 2004) that catalyze the formation of the Zn chelator nicotianamine (NA) that is important for Zn distribution. The TFs themselves are typically embedded in a negative feedback loop, which ensures that Zn sufficiency downregulates F-bZIP activity (Figure 4).

In *A. thaliana*, bZIP19 and bZIP23 TFs are important regulators of genes involved in Zn homeostasis (Assunção et al., 2010) and close homologues have been identified in rice (OsbZIP48 and OsbZIP50; Lilay et al., 2019), wheat (TabZIPF1 and TabZIPF4; Evens et al., 2017), and barley (HvbZIP56 and HvbZIP62; Nazri et al., 2017). Functionally, the bZIP19 and bZIP23 are partially redundant, with single knock-out mutants only showing very subtle phenotypes under Zn deficiency conditions (Assunção et al., 2010, 2013). However, the double mutant *bzip19/23* shows severe growth inhibition and chlorosis under Zn deficiency.

Studies on *A. thaliana* F-bZIPs have greatly helped understanding their molecular mechanism. Zn-reporting bZIPs contain a highly conserved region that is rich in cysteine and histidine residues. It is located in the N-terminal region, upstream of the bZIP domain that is important for DNA binding and dimerization. The Cys/His rich motif is involved in Zn binding (Assunção et al., 2013) and thus acts as a zinc sensor motif (ZSM) (Lilay et al., 2021). Zn binding to *Arabidopsis* bZIP19 and bZIP23 was demonstrated directly by carrying out *in vitro* binding assays after bZIP expression in *E. coli*. Incubation of bZIP19 and bZIP23 proteins with stable isotope (^65^Zn) followed by analysis with size exclusion chromatography and inductively coupled plasma mass spectrometry showed that each TF molecule binds two Zn atoms (Lilay et al., 2021). *In vivo*, the TFs would presumably bind the free Zn^2+^ within the nucleus to each monomer when Zn is present in sufficient amount. This inactivates the TFs, most likely via conformational changes that prevent TF binding to the DNA (Lilay et al., 2021). In the absence of Zn^2+^, the ZSM is free of Zn and the TF conformation favors DNA binding. More details on the molecular mechanisms of Zn binding, for example, using crystal structures, could help us understand the role of conformational changes and how they impact TF functionality.

It is likely that alternative, bZIP-independent, Zn sensing mechanisms exist. For instance, the promoters of the *Arabidopsis* ZIP genes are different with respect to the number, arrangement, and nucleotide sequence of ZDRE elements (Castro et al., 2017). Whether these aspects are directly correlated to the ZIP expression pattern under Zn deficiency is still to be resolved. For example, the rice *ZIP4* gene contains no ZDRE element in its promoter region, yet its expression is dependent on Zn levels (Castro et al., 2017). Regulation of OsZIP4 and similar transporters may be post-translational, as has been found in mammalian cells where ZIP7 activity is modulated by phosphorylation (Taylor et al., 2012).

Though the above shows that impressive progress was achieved regarding Zn deficiency responses, the mechanism(s) to restrict excess Zn are less well understood. Removal of Zn from the cytosol is largely mediated by Metal Tolerance Proteins (MTPs), members of the ubiquitous Cation Diffusion Facilitator (CDF) family. Specific MTP isoforms move Zn into the vacuole (Arrivault et al., 2006; Desbrosses-Fonrouge et al., 2005), the ER (Sinclair et al., 2018), or Golgi (Fujiwara et al., 2015). The tonoplast localized MTPs transport Zn^2+^ into the vacuole when its cytoplasmic concentration exceeds optimum values. In *A. thaliana*, this is carried out by the ubiquitously expressed AtMTP1 (Desbrosses-Fonrouge et al., 2005). *AtMTP1* transcript is not dependent on Zn levels. Instead, AtMTP1 activity may be regulated at the protein level by the cytosolic Zn levels (Tanaka et al., 2015); AtMTP1 contains a histidine-rich cytoplasmic loop between transmembrane domain 4 and 5 that can bind Zn and hence acts as a sensor that reports on cytoplasmic Zn levels (Kawachi et al., 2008; Tanaka et al., 2015). Isothermal titration calorimetry showed that the AtMTP1 His-rich loop binds four Zn^2+^ per monomer *in vitro* (Tanaka et al., 2015). As described above for bZIPs, Zn binding to the His-rich domain is likely to induce conformational changes that regulate transporter activity (Tanaka et al., 2015), making MTP1 a tonoplast located transceptor. This notion confirmed earlier hypotheses about the role of the AtMTP1 His-rich loop in MTP activity, which was based on homology structural models of the plant (Podar et al., 2012) and *E. coli* CDFs (Lu and Fu, 2007; Lu et al., 2009). Similar to AtMTP1, AtMTP2 and AtMTP3 possess cytoplasmic loops between transmembrane domains 4 and 5 with the potential to bind Zn and act as Zn sensors.

Efflux of Zn to the apoplast is another important constituent of Zn homeostasis that typically relies on the activity of heavy metal ATPases (HMAs) (Argüello, 2003; Hanikenne et al., 2008; Hussain et al., 2004; Mills et al., 2012; Morel et al., 2009; Siemianowski et al., 2011; Sinclair et al., 2018; Verret et al., 2005; Williams et al., 2000). Efflux not only removes excess Zn, but also provides Zn for translocation to shoots and Zn remobilization such as deposition of Zn in seeds. Such processes must be integrated into overall Zn homeostasis and are likely to include dedicated Zn sensors but the identity of the latter is unknown. *A. thaliana* HMA4 is one of the main drivers of Zn root to shoot translocation. It possesses a C-terminal domain containing 13 cysteine and a stretch of 11 His that were proposed to be involved both in binding and sensing of Zn and Cd ions (Baekgaard et al., 2010).

### Copper (D)

Copper (Cu) is essential as a constituent of mitochondrial and photosynthetic redox chains and as cofactor of many crucial enzymes such as superoxide dismutases. Excess Cu, like Fe (Yruela, 2009), is highly toxic because free Cu^+^ catalyzes the production of free radicals. In plants, there is good evidence that Cu^2+^ uptake and distribution are regulated via the transcription factors belonging to the Squamosa promoter binding protein (SBP) family that has only been found in plants. SBP proteins contain an SBP-domain consisting of 76 amino acids that include a nuclear localization signal (NLS) and 8 conserved Cys and His residues arranged in two atypical zinc finger structures (a C-C-CH motif for the first and C-C-H-C motif for the second zinc finger) (Sommer et al., 2010; Yamasaki et al., 2004, 2006). Binding of SBPs to DNA occurs at GTAC promoter sequences that are found in copper response elements (CuREs) of target genes. Mutation of any of the nucleotides in the GTAC motif impairs TF binding and additional sequences apart from the core GTAC are required for the initiation of transcription (Nagae et al., 2008; Quinn et al., 2000; Yamasaki et al., 2009).

The genome of the green alga *Chlamydomonas reinhardtii* encodes 24 SBPs of which Copper response regulator (CRR1) is a key Cu dependent SBP-TF that activates more than 60 genes during Cu deficiency (Yamasaki et al., 2004). Target genes include Cu^+^ transporters from the CTR family, and genes that encode cytochrome c6 (a photosynthetic electron carrier that replaces plastocyanin when Cu is deficient). CRR1 contains a Cys-rich C-terminal region similar to the Cu^+^ sensing domain of the metal-responsive factor-1 of *Drosophila melanogaster* (Chen et al., 2008). Binding studies revealed that in addition to the zinc finger regions, CRR1 has two highly conserved Cys/His residues that are needed for Cu binding and that Cu^+^, rather than Cu^2+^, is the preferred liganded metal. Although *in vitro* studies showed that Zn^2+^ can displace Cu^+^ from the protein, *in vivo*, Cu^+^ is more likely to be the bound metal owing to a much greater Cu^+^ affinity of the protein. The greater selectivity for Cu^+^ combined with reversible high-affinity Cu^+^ binding implies that SBP proteins like CRR1 are TFs that can function as primary sensors of copper. In agreement with this notion is the observation that in the Cu^+^-bound form CRR1 does not interact with DNA (Merchant et al., 2020) and only when Cu^+^ is depleted do these TFs bind to the promoter region of Cu-regulated genes. How changes in Cu status are translated into transcriptional adjustment has yet to be unraveled; nuclear CRR1 localization could respond to altered Cu (Merchant et al., 2020; Sommer et al., 2010). Alternatively, Cu binding to the CRR-SPB domain may cause conformational changes that affect the CRR-DNA interaction (Sommer et al., 2010). This latter hypothesis stems from electrophoretic mobility shift assays that show that Cu-bound CRR1 does not bind to DNA (Sommer et al., 2010).

Higher plants contain many SPBs which are called Squamosa promoter binding protein like (SPLs) (Birkenbihl et al., 2005; Chen et al., 2010; Zhou et al., 2018). They are extremely diverse and share little amino acid sequence homology apart from the SPB domain. The *A. thaliana* genome encodes around 17 SPLs (Birkenbihl et al., 2005; Liang et al., 2008) with SPL7 being the closest ortholog of CRR1 (Bernal et al., 2012; Schulten et al., 2019; Yamasaki et al., 2009). SPL7 activity has been found to promote high-affinity Cu-uptake and to optimize Cu (re-)distribution to essential Cu-proteins (Bernal et al., 2012). This regulatory pathway is based on specific miRNAs that target mRNA that encodes dispensable Cu proteins. An example is the degradation of Cu containing super oxide dismutases (SODs) and their replacement with iron- or manganese-SODs, in order to preserve Cu (Schulten et al., 2019).

Though implicated in Cu homeostasis, SPL7 is unlikely to function as a primary Cu sensor because its transcript levels are largely non-responsive to Cu availability (Garcia-Molina et al., 2014). Furthermore, in the presence of Cu, SPL7 does not bind to DNA *in vitro* (Sommer et al., 2010). Compared with *C. reinhardtii*, the cellular SPL7 location is also different (Garcia-Molina et al., 2013, 2014; Sommer et al., 2010); whereas CRR1 is exclusively expressed in the nucleus, GFP-tagged SPL7 showed it is associated with the ER via a C-terminal transmembrane domain that is absent in the algal CRR1 (Garcia-Molina et al., 2014). Thus, Cu sensing *per se* and regulation of SBP-TFs in higher plants is likely to be different from the algal system and an, as yet unknown, sensor functions upstream of SPLs. Instead, the role of SPLs in higher plant Cu homeostasis may be indirect and based on post-translational modification (Garcia-Molina et al., 2020). The latter notion comes from work that showed SPL7 interacts with a KIN17-domain protein, an interaction that led to raised SPL7 activity and increased transcription of target genes during Cu deprivation (Garcia-Molina et al., 2014).

In all, there is good evidence that Cu sensing in lower plants is carried out by members of the SPB family, whereas in higher plants, SPBs do not respond to Cu and hence are unlikely to function as the primary sensor. This implies that an unknown component fulfills the sensing role. Alternatively, SPLs may not respond to (free) ionic copper, the concentration of which is exceedingly low, but interact with Cu that is complexed by organic factors such as metallothioneins (Garcia-Molina et al., 2020).

### Iron

Iron (Fe) is found in many enzymes and its transition metal properties make it an ideal cofactor in many redox reactions. Consequently, it is a major constituent of electron transfer chains in mitochondria and chloroplasts. Furthermore, it is essential for the biosynthesis of chlorophyll and thus pivotal for photosynthesis. In contrast, free Fe^2+^ (like free Cu^+^) can be extremely toxic as it is a potential source of oxygen radicals.

Fe is the second most abundant metal in the earth’s crust but is predominantly present as highly insoluble oxides and plants therefore had to evolve potent mechanisms to extract Fe from the soil. Two main strategies emerged: in dicotyledonous and nongramineous monocots, Strategy I ensures solubilization of Fe in the rhizosphere via acidification by H^+^ releasing ATPases and the reduction of Fe^3+^ to the more soluble Fe^2+^ by FRO-type Fe(III)-chelate reductases. Fe^2+^ is subsequently taken up across the plasma membrane by Fe^2+^ transporters of the iron-regulated transporter 1 (IRT1) family. Strategy II, used by gramineous monocots, is based on the excretion of high-affinity Fe chelators in the form of phytosiderophores that consist of mugineic and deoxymugineic acids (Nozoye et al., 2011, 2015). Phytosiderophores bind and solubilize Fe^3+^ and the Fe-chelator complex is then transported into the root symplast via Yellow Stripe (YS) or YS-like (YSL) transporters (Curie et al., 2001).

Fe homeostasis is maintained through intricate signaling pathways that rely on the contribution of various classes of proteins.

#### HRZ-BTS-BTSLs proteins

**Figure 5:**
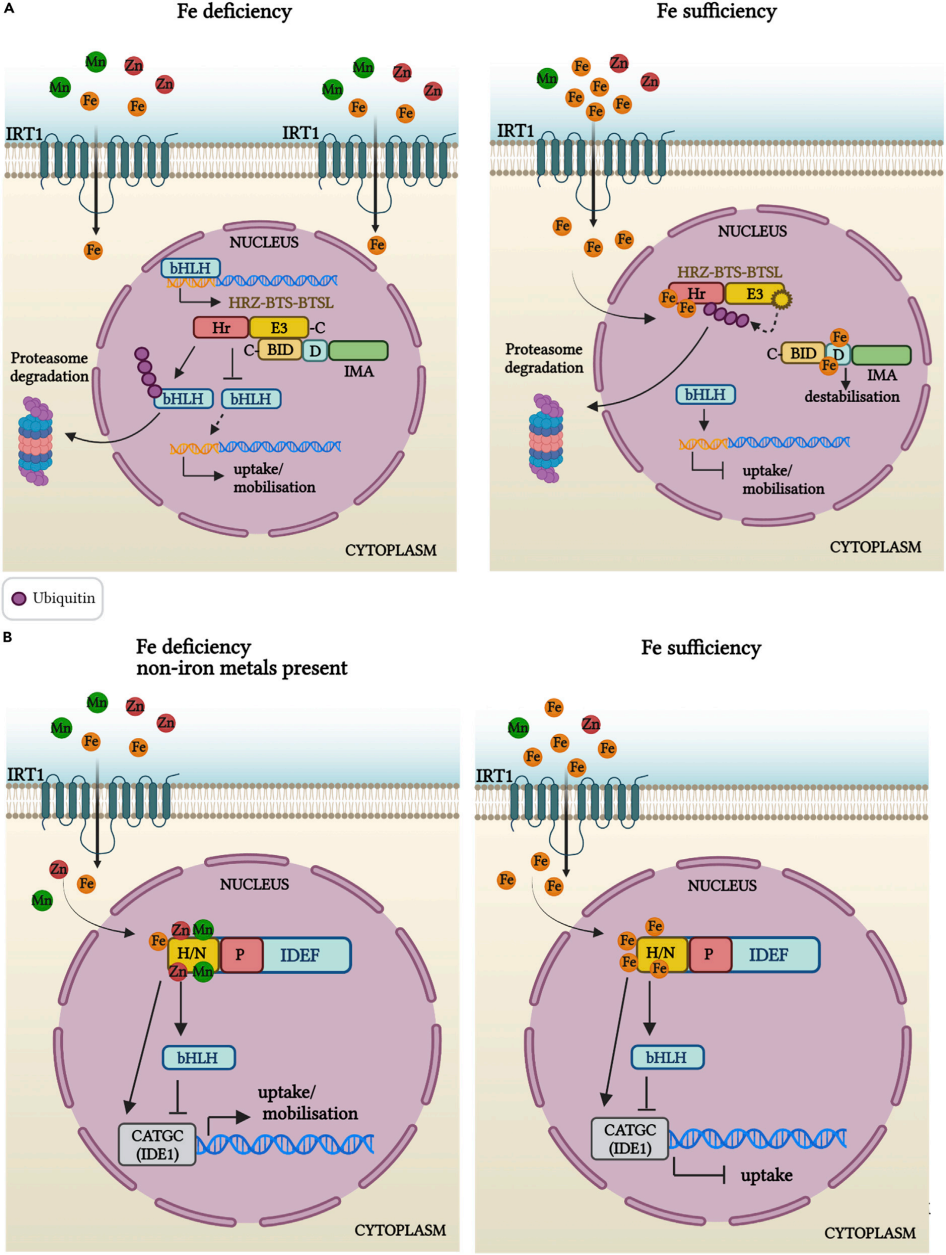
Iron sensing and homeostasis HRZ-BTS-BTSL regulation of Fe homeostasis. (A) (Left panel) In Fe deficiency conditions, Fe-deficiency response genes are induced and thus uptake and mobilization of Fe occurs. Among the upregulated genes are members of the HRZ-BTS-BTSL families with HRZ-BTSs mostly found in the root stele and shoots and BTSLs found only in the root rhizodermis, cortex, and endodermis of dicotyledonous species. Stabilization of HRZ-BTS-BTSL requires low amounts of Fe, and allows their interaction with bHLH TFs that are positive regulators of Fe uptake and mobilization while, at the same time, promoting HRZ-BTS-BTSL degradation, thus preventing Fe overload. Within the root stele and shoots, HRZ-BTSs can further interact with IMA-FEP peptides whose expression is induced by Fe deficiency. IMA-FEPs bind through their C-terminal binding domain (BID) to the HRZ-BTS, thus preventing HRZ-BTS interactions with bHLHs TFs. (Right panel) In Fe-sufficient conditions, HRZ-BTS-BTSL can directly bind Fe^2+^ or other non-iron ions at their Hr motifs that cause destabilization of the protein by autoubiquitination via its C-terminal E3 ligase domain and subsequent degradation. IMAs are also destabilized through Fe binding to an aspartate-rich sequence and this similarly blocks bHLH-mediated Fe uptake and mobilization. (B) IDEFs are TFs that are found in graminaceous species and present in all tissues, independent of the Fe status. IDEFs regulate the response to Fe deficiency in rice by recognizing CATGC sequences within the Fe deficiency-responsive, cis-acting element IDE1 (left panel). In Fe sufficiency or the presence of other metals, IDEF can bind Fe (or other divalent metals such as Cu, Mn, Ni, and Zn) to the N-terminal histidine–asparagine (N/H) and proline-rich (P) tandems of a designated metal binding site. This impedes IDEF interaction with bHLH TFs that positively regulate metal uptake and mobilization (right panel). HRZ-BTS-BTSLs proteins, haemerythrin motif-containing Really Interesting New Gene (RING)- and Zinc-finger proteins/BRUTUS/BRUTUS(-like) [BTS(L)]; bHLH, basic helix-loop-helix; TF, transcription factor; IMA, iron man; FEP, Fe uptake-inducing peptide; BID, binding domain; Hr, haemerythrin; D, aspartate-rich sequence; IDEFs proteins, iron deficiency-responsive element-binding factors; N/H, histidine–asparagine; P. proline. Figure created with BioRender.com.

Haemerythrin motif-containing Really Interesting New Gene (RING)- and Zinc-finger proteins from the HRZ/BRUTUS-like (BTSL) family are key regulators of Fe signaling and are proposed to act as primary Fe sensors. HRZ-BTSs are present in all green plants, whereas BTSLs are present only in dicotyledonous species like *A. thaliana* (Kobayashi et al., 2013; Rodríguez-Celma et al., 2019). HRZ-BTS-BTSLs are nuclear proteins (Selote et al., 2015) and contain an N-terminal haemerythrin (Hr) domain and a C-terminal RING-type E3 ubiquitin ligase (Hindt et al., 2017; Kobayashi et al., 2013; Long et al., 2010; Rodríguez-Celma et al., 2019; Selote et al., 2015). In analogy to mammals, the HRZ-BTS Hr domain has been proposed to function as a cellular Fe sensor (Rodríguez-Celma et al., 2019; Ruiz and Bruick, 2014). In mammalian cells, Hr domains fold in a bundle of four α-helices that contains two Fe atoms (each coordinated by histidine and acidic residues). The necessary conformational changes that cause folding or de-folding depend on the Fe concentration; in Fe-sufficient conditions, the Hr domain is stabilized and resistant to proteolysis allowing its accumulation within the cell (Ruiz and Bruick, 2014). In Fe-deficient conditions, the Hr di-iron center is compromised, allowing HRZ ubiquitination and subsequent degradation by E3 ligases (Ruiz and Bruick, 2014). In contrast to the Hr Fe sensor in mammals which is stabilized in the presence of Fe, in plant cells Fe binding to the N-terminal of Hr domains of BTS drives protein destabilization (Selote et al., 2015). Furthermore, Fe sensing by HRZ-BTS-BTSL and the subsequent regulation mechanisms may be more complex in plant cells owing to the presence of multiple Hr domains and the presence of C-terminal E3 ligases that can catalyze self-ubiquitination (Kobayashi et al., 2013; Matthiadis and Long, 2016; Rodríguez-Celma et al., 2017, 2019; Selote et al., 2015, 2018). HRZ-BTSs and BTSLs have dissimilar numbers of Hr motifs and have different localization within the plant with HRZ-BTSs located in the root stele and shoots (Dinneny et al., 2008; Kobayashi et al., 2013; Long et al., 2010), whereas BTSLs are restricted to the root rhizodermis, cortex, and endodermis (Hindt et al., 2017; Rodríguez-Celma et al., 2017). In light of these differences, HRZ-BTSs were proposed to act on Fe mobilization and BTSLs on uptake of Fe (Rodríguez-Celma et al., 2019). HRZ-BTS-BTSL proteins negatively regulate Fe homeostasis by promoting the ubiquitin-mediated degradation of bHLH TFs that positively regulate the Fe deficiency response (Gollhofer et al., 2014; Hindt et al., 2017; Long et al., 2010; Selote et al., 2015; Sivitz et al., 2011; Rampey et al., 2006; Rodríguez-Celma et al., 2017, 2019) (Figure 5A). However, it is important to note that, although HRZ-BTS-BTSLs act as negative regulators of Fe-deficiency induced genes, their expression is also induced under Fe deficiency by the same bHLH TFs that are ubiquitinating. Since the translation of HRZ-BTS-BTSLs requires small amounts of Fe, there is a delay in the feedback loop that induces the HRZ-BTS-BTSLs accumulation. This delay allows other Fe-related proteins to replenish Fe before HTZ-BTS-BTSLs ubiquitinate bHLHs. The raise in Fe causes destabilization and degradation of HRZ-BTS-BTSLs to a residual level required for fine-tuning the amount of Fe within the plants, thus preventing toxic overload (Rodríguez-Celma et al., 2017, 2019; Selote et al., 2015, 2018). Evidence for the above notion comes from *bts* knockout mutants that are embryo lethal because they accumulate toxic levels of Fe in the seeds (Selote et al., 2015). Furthermore, HRZ-BTS (but not BTSL) activity is also regulated through their interaction with IMA-FEPs described below.

#### IDEF transcription factors

Iron deficiency-responsive element-binding factors (IDEFs) are TFs that are present in rice and barley but homologues have not been identified in non-graminaceous species. *OsIDEF1* and *OsIDEF2* transcript levels are independent of Fe status and can be detected in all plant tissues (Kobayashi et al., 2007, 2009, 2010; Ogo et al., 2008). However, OsIDEF1 and HvIDEF1 can bind Fe^2+^ (or other divalent metals such as Cu, Mn, Ni, and Zn) to histidine–asparagine and proline-rich tandem metal-binding site in their N-termini (Kobayashi et al., 2012). IDEFs regulate the response to Fe deficiency in rice by recognizing CATGC sequences within the Fe deficiency-responsive, cis-acting element IDE1 (Kobayashi et al., 2009). Moreover, the level of metal binding directly correlates with the amount of metals in the solution (Kobayashi et al., 2012). This suggests IDEFs could function as primary sensors that use metal binding to the His-Asn repeats to report on Fe status (Figure 5B).

#### IMA-FEP proteins

Iron man-Fe-uptake inducing peptides (IMA-FEPs) are present in all angiosperms and were put forward as another class of proteins that acts in Fe signaling and sensing (Grillet et al., 2018; Hirayama et al., 2018; Kobayashi et al., 2020). During Fe deficiency, IMA-FEP levels increase in the phloem, i.e. in companion cells of sieve elements, and thus these peptides are able to circulate throughout the plant (Grillet et al., 2018; Hirayama et al., 2018). IMA-FEPs can interact with HRZ-BTSs; the IMA-FEP C-terminal contains a conserved BTS interaction domain (BID) that is responsible for their association with the C-terminal of BTS. The interaction prevents binding of BTSs to bHLH TFs (see above) that are responsible for inducing a plant response to Fe deficiency (Li et al., 2021). In addition, the relevant bHLH TFs also contained BIDs allowing the IMA-FEPs to stabilize bHLH proteins by competitively interacting with BTS (Li et al., 2021). IMA-FEPs can directly bind Fe, Mn, Cu, and Zn to a conserved C-terminal aspartate-rich 17 amino acid sequence (Grillet et al., 2018; Kobayashi et al., 2020). Furthermore, Fe sufficiency triggers IMA-FEPs destabilization, as shown by *in vitro* studies where saturation of the binding sites caused IMA-FEPs precipitation (Grillet et al., 2018). Such results suggest that IMA-FEPs may act as (phloem) sensors but further experimental results are needed to support this notion.

#### IRT1 transporters

One of the main players in Fe uptake is IRT1, a primary Fe^2+^ uptake system localized to the plasma membrane of root rhizodermal cells (Dubeaux et al., 2018). Whereas Fe is the primary substrate of IRT1 it also drives the movement across the membrane of other metals such as Mn, Zn, Co, and Cd (Barberon et al., 2011, 2014; Eide et al., 1996; Korshunova et al., 1999; Rogers et al., 2000; Vert et al., 2002, 2003).

IRT1 transport activity is tightly regulated both at the transcriptional and posttranscriptional level depending on the concentration of Fe and other metals. Transcriptional regulation of *IRT1* is orchestrated by a complex network of bHLH TFs (from subgroup Ib and IVb). In Fe-deficient conditions, transcription of IRT1 is upregulated by binding of heterodimers that consist of bHLH TFs and FIT (Fe-deficiency-induced transcription factor) to the *IRT1* promoter (Colangelo and Guerinot, 2004; Eide et al., 1996; Jakoby et al., 2004; Lei et al., 2020; Vert et al., 2002; Yuan et al., 2005). Both local root signals and systemic signals impact on transcriptional regulation of *IRT1* (Grusak and Pezeshgi, 1996).

**Figure 6:**
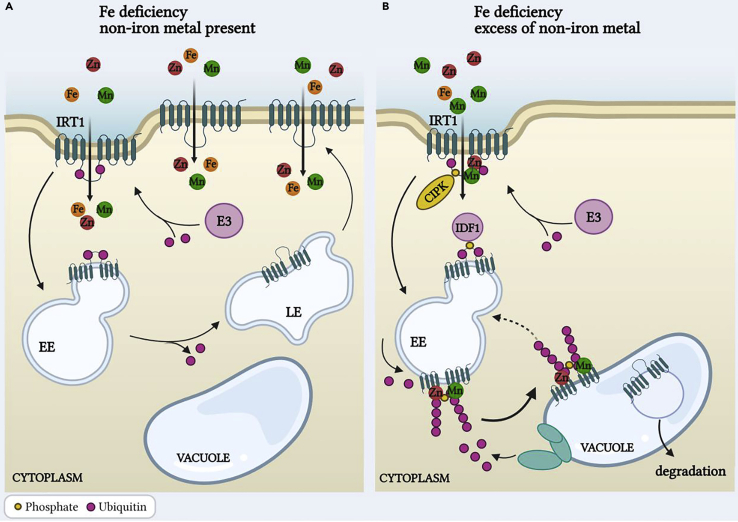
Transceptor function of IRT1 (A) IRT1 functionality is normally balanced by an endocytotic degradation pathway (via E3 ligase ubiquitination) to early endosomes and vacuole, and retrograde trafficking back to the plasma membrane via late endosomes. (B) In the presence of excess non-iron metals, IRT1 functions as a transceptor that down-regulates metal uptake; non-iron metals bind to the cytoplasmic histidine-rich loop of IRT1, which causes IRT1 phosphorylation by the CIPK23 and subsequent multimonoubiquitination by E3 ligase. Ubiquitination promotes endocytosis and vacuolar degradation via the trans Golgi/early endosomes. IRT1, iron regulated transporter; EE, early endosomes; LE, late endosomes; CIPK23, CBL-interacting serine/threonine-protein kinase 23. Figure created with BioRender.com.

Post-transcriptionally, the activity of IRT1 is modulated through trafficking between plasma membrane and endosomes (Ivanov et al., 2014) (Figure 6A). However, this is largely independent of the Fe status and more relevant in preventing the accumulation of non-iron metals (Barberon et al., 2011). Excess of non-iron metals (e.g. Mn, Zn, Co, or Cd) causes their binding to the cytoplasmic histidine-rich loop of IRT1 (Dubeaux et al., 2018). Binding of non-iron metal induces IRT1 phosphorylation at neighboring serine and threonine amino acids by the CBL-interacting serine/threonine-protein kinase 23 (CIPK23) and subsequent multimono-ubiquitination (Barberon et al., 2011; Kerkeb et al., 2008; Shin et al., 2013). Ubiquitination activates endocytosis of the transporter to the trans Golgi/early endosomes and designates IRT1 protein for vacuolar degradation by E3 ligases (Dubeaux et al., 2018; Shin et al., 2013) (Figure 6B).

The multimono-ubiquitination-mediated endocytosis of IRT1 is an important protective mechanism to prevent excess of non-iron metals such Mn, Zn, Co, or Cd. In this respect, IRT1 functions as a transceptor that transports Fe and Zn, Mn, or Co, whereas it directly senses and reports on excess of Zn, Mn, and Co ions in the cytoplasm (Cointry and Vert, 2019; Dubeaux et al., 2018).

In all, there is as yet no conclusive evidence regarding primary sensors for Fe status. The data point to multiple candidates, with at least one acting as a transceptor (IRT1), whereas several others consist of cytosolic or nuclear proteins with clearly defined metal-binding sites (e.g. HRZ-BTS-BTSL proteins, IDEF TFs) albeit often with multiple metal selectivities. Peptides may play important roles in the sensing and signaling of Fe status between root and shoot.

### Other micronutrients

Currently there is no information about plant sensors that report on Mn, Ni, B, or Mo. Information about sensing of these nutrients (Mn and Ni) comes primarily from work on bacteria. For example, Mn sensors in bacteria have been characterized and may involve both protein type sensors (i.e. transcription factors that bind Mn) and RNA-based sensors (riboswitches) that regulate the expression of Mn transporters (Waters, 2020). These sensors regulate Mn uptake systems such as ABC and NRAMP transporters. For Ni, bacteria such as *E. coli* actively import this element via ABC transporters that are under control of Ni binding transcription factors like NikR (Chivers and Sauer, 2000). NikR has a Ni binding affinity in the picomolar range.

## Conclusions and outlook

Nutrients are often scarce in the environment, which generates considerable evolutionary pressure to develop mechanisms to optimize nutrient use efficiency. A crucial part of this is the monitoring of external and internal nutrient levels, which, for 16 essential nutrients, would require a minimum of 32 primary sensors. This number would be considerably larger if we further consider that multiple internal sensors may be needed to accommodate the monitoring of nutrient levels in various cellular compartments and in different organs. From our evaluation, it is clear that as yet only very few of these have been identified but steady progress is being made using a range of methodologies.

A broader perspective across all kingdoms suggests that there are two main mechanisms employed by primary sensors, transceptors, and transcription factors. Transporters that also act as receptors are emerging as a common theme in the regulation of nutrient transport by sensing local nutrient concentrations. Examples include NRT1; 1 for nitrate and IRT1 for a range of divalent nutrients. For TFs, a mutational approach can be highly lucrative and has been applied very successfully especially in microorganisms to identify sensors. The identification of the Fur Fe sensor of *E. coli* is a case in point (Hantke, 1987). In the case of metal nutrients, such screens can be informed by sequence information about the presence of metal-binding domains such as clusters of cysteine or histidine amino acids. Site-directed mutational assays can help establish affinity and selectivity of metal binding and its impact on the TF-DNA interaction. Looking for changes or abolition of early signals, such as the salinity-induced Ca signal, is another strategy that may identify upstream primary sensor components. In contrast, confirmation of transceptor function is more complicated because of overlap and pleiotropic effects that confound separating transport and signaling events. This is particularly the case for multisubstrate transporters such as ZIP1. Extensive mutational screens may be necessary to identify residues and functional protein domains that uncouple the transport and signaling functions.

Combined nutrient stress may occur in many soil types and optimization also critically depends on nutrient interactions such as the necessity to balance nutrients for overall biosynthesis and development, or charge balancing of cations and anions. Some of these interactions could be associated with primary sensors as is the case for IRT1 that not only functions as an Fe transporter but also as a transceptor for Zn, Mn, and Co. The putative TF-based Cu sensor CRR1 of *Chlamydomonas* has a Cys-rich C-terminal region that forms a ligand to both Cu and Zn; association of Zn to this part of CRR1 led to a five-fold induction of multiple ZRT Zn transporters (Sommer et al., 2010). Lipid-based sensors as described for Na are in essence monovalent cation reporters so could also function in response to alterations of ambient K, Li, or Cs levels (Jiang et al., 2019). In other cases, nutrient homeostatic mechanisms are more likely to be integrated at further downstream signaling steps. This is exemplified by the hub function of the CIPK23 kinase, which is involved in the regulation of multiple nutrients and appears to coordinate responses to low potassium, low nitrate, low iron, high ammonium, and high magnesium (Ródenas and Vert, 2021).
